# Extracting psychiatric stressors for suicide from social media using deep learning

**DOI:** 10.1186/s12911-018-0632-8

**Published:** 2018-07-23

**Authors:** Jingcheng Du, Yaoyun Zhang, Jianhong Luo, Yuxi Jia, Qiang Wei, Cui Tao, Hua Xu

**Affiliations:** 1 The University of Texas School of Biomedical Informatics, 7000 Fannin St Suite 600, Houston, TX 77030 USA; 2 Department of Management Science and Engineering, Zhejiang Sci-Tech University, Hangzhou, 310018 China; 3 Department of Medical Informatics, School of Public Health, Jilin University, Changchun, 130021 Jilin China

**Keywords:** Suicide, Mental health, Psychiatric stressors, Social media, Deep learning, Named entity recognition

## Abstract

**Background:**

Suicide has been one of the leading causes of deaths in the United States. One major cause of suicide is psychiatric stressors. The detection of psychiatric stressors in an at risk population will facilitate the early prevention of suicidal behaviors and suicide. In recent years, the widespread popularity and real-time information sharing flow of social media allow potential early intervention in a large-scale population. However, few automated approaches have been proposed to extract psychiatric stressors from Twitter. The goal of this study was to investigate techniques for recognizing suicide related psychiatric stressors from Twitter using deep learning based methods and transfer learning strategy which leverages an existing annotation dataset from clinical text.

**Methods:**

First, a dataset of suicide-related tweets was collected from Twitter streaming data with a multiple-step pipeline including keyword-based retrieving, filtering and further refining using an automated binary classifier. Specifically, a convolutional neural networks (CNN) based algorithm was used to build the binary classifier. Next, psychiatric stressors were annotated in the suicide-related tweets. The stressor recognition problem is conceptualized as a typical named entity recognition (NER) task and tackled using recurrent neural networks (RNN) based methods. Moreover, to reduce the annotation cost and improve the performance, transfer learning strategy was adopted by leveraging existing annotation from clinical text.

**Results & conclusions:**

To our best knowledge, this is the first effort to extract psychiatric stressors from Twitter data using deep learning based approaches. Comparison to traditional machine learning algorithms shows the superiority of deep learning based approaches. CNN is leading the performance at identifying suicide-related tweets with a precision of 78% and an F-1 measure of 83%, outperforming Support Vector Machine (SVM), Extra Trees (ET), etc. RNN based psychiatric stressors recognition obtains the best F-1 measure of 53.25% by exact match and 67.94% by inexact match, outperforming Conditional Random Fields (CRF). Moreover, transfer learning from clinical notes for the Twitter corpus outperforms the training with Twitter corpus only with an F-1 measure of 54.9% by exact match. The results indicate the advantages of deep learning based methods for the automated stressors recognition from social media.

## Background

Suicide has been one of the leading causes of deaths in the United States [1]. An average of 44,965 Americans die by suicides each year [2]. The national cost of suicides and suicidal behavior in the United States was $93.5 billion in 2013 after adjustment for under-reporting [3]. According to the National Institute of Mental Health, the total suicide rate has increased 24% over the past 15 years [4]. Suicide and suicidal behaviors not only cause unbearable impacts on the specific individuals and surviving family and friends, but also create long lasting effects on whole communities [5].

One of the first steps toward suicide prevention is the identification of risk factors and causes associated with suicide [6].The multiple causes of suicide and suicidal behaviors can be broadly divided into stressors or triggers and predisposition [7]. As one of the major causes to suicide, psychiatric stressors are psychosocial or environmental factors that can profoundly impact cognition, emotion, and behavior of people [8]. The causes of suicide and suicidal behaviors can be complex and vary greatly from individual to individual. The identification of psychiatric stressors is critical to understanding the causes of potential suicide and suicidal behaviors for a specific individual, which is critical to provide a tailored and precise intervention strategy. For example, if the primary stressor for an individual is identified as school bullying, we can offer intensive individual interventions that provide the victim with individual support through meetings with students and parents, counseling, and sustained child and family support [9].

Mentions of stressors are often embedded in narratives such as clinical text or social media posts, and thus need to be recognized first for further investigation. Fortunately, the advances in machine learning and natural language processing (NLP) provide great opportunities to access mental health issues from large-scale narrative data. For example, Zhang et al. mined Electronic Health Records (EHR) to extract psychiatric stressors and symptoms from clinical text [8, 10]. In recent years, social media has shown significant value for many public health related issues [11], as well as the influence on mental health and suicide-related behaviors [12, 13]. The wide popularity of social media provides unprecedented opportunities to access mental health and suicide risk from a large-scale population. The real-time information sharing flow on social media allows potential early detection and intervention for at-risk users.

Most previous studies focused on analyzing the association between suicidal ideation and the linguistic features of the contents (i.e. lexical analysis [14–18]) or posting behaviors (i.e. posting frequency [5, 19]) on social media platforms. Some recent efforts attempted to classify tweets by levels of distress [6], concerns [20, 21] or types of suicidal communication [22] using machine learning or rule based approaches. However, few studies have been done mining the risk factors from social media. Jashinsky et al. tracked suicide risk factors from Twitter using keywords-based approaches [5]. We explored the association between psychiatric stressors and symptoms in tweets based on domain terminologies using Elasticsearch in the paper published in BIBM SEPDA 2017 workshop [23]. However, the keywords-based approaches often retrieve many irrelevant tweets, which introduce much noise into the further analyses.

As an extension to the conference paper, we narrowed down the focus of this paper to automatic identification of suicide related psychiatric stressors and propose a complete deep learning based pipeline to extract psychiatric stressors for suicide from Twitter data. This pipeline first collected and filtered suicide related tweets using keywords. Then, a convolutional neural network (CNN) based classifier removed more noise by further filtering out irrelevant tweets. After that, we applied a recurrent neural network (RNN) based algorithm to extract the mentions of stressors from texts of the suicide related tweets. Furthermore, given that it is time-consuming and costly to build an annotation dataset of stressors in tweets, we also examined the impact of transfer learning based approaches by leveraging pre-trained neural network layers training on clinical notes. To the best of our knowledge, it is the first effort to use systematic machine learning based approaches to extract psychiatric stressor from social media. It could have significant impacts on how people identify those in need of mental health services such as suicide prevention.

## Methods

### System overview

**Fig. 1:**
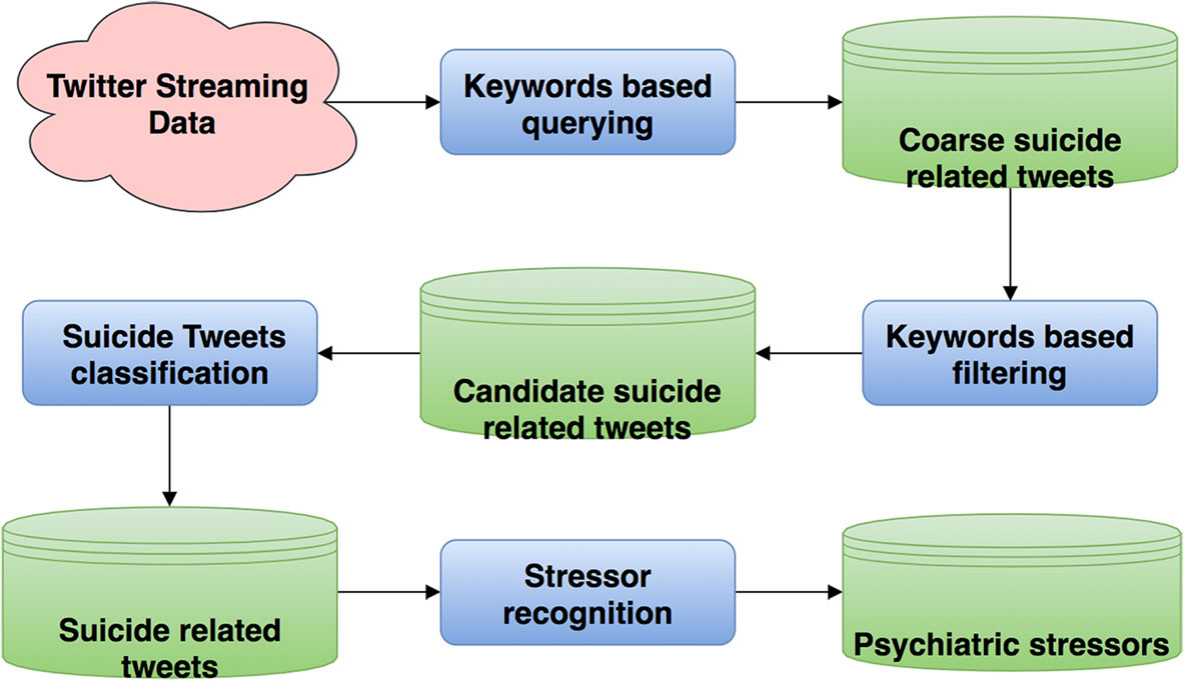
Pipeline of suicides related psychiatric stressor recognition from Twitter

As illustrated in Fig. 1, the pipeline of the psychiatric stressor recognition from Twitter consisted of four steps. First, we retrieved a coarse set of suicide-related tweets using suicide-related keywords. Here we define the suicide related tweets as the tweets that contain the potential suicidal ideations, suicide history or plan etc. for the Twitter users. Second, we generated a refined candidate set of suicide related tweets by filtering tweets with the obvious stop words collected manually. Third, considering that keywords based tweets collection will introduce much noise, we employed a deep learning based classification model to further select out the precise suicide related tweets. Specifically, the CNN based approach was employed in this step. Finally, we detected the mentions of psychiatric stressors from the suicide-related tweets generated from the previous step. This problem was conceptualized as a named entity recognition (NER) task and we leveraged a state-of-art RNN based framework for this step. In addition, we further investigated transfer learning strategy to improve the performance of stressor extraction.

### Tweets collection and filtering

We manually curated a suicide related keywords/phrases list to collect public suicide related tweets from June 26th, 2017 to Oct. 19th, 2017 through Twitter streaming API. The keywords/phrases list contains 21 keywords/phrases, such as “suicide”, “kill myself”, “want death”, etc. While manually reviewing the collected tweets, we found most of the tweets were discussing news or advertisements instead of personal ideation or experience. It would have created higher annotation burden and would have deteriorated the performance of the machine learning system if we used the collected tweets directly. To create a refined candidate set of more precise suicide related tweets, we removed all the tweets that contained an URL or keywords such as “hotline”, “suicide bomb”, “suicide attack”, etc. during the collection period. We inductively generated the stop words list to filter the tweets. By doing so, we observed a strong increase in the relevant tweets, which is beneficial for the training and evaluation of the deep learning system. The full list of keywords and stop keywords can be seen in Table 1. After filtering, 1,962,766 tweets were collected during this time period.

**Table 1:** Examples of suicide-related keywords as queries for tweets retrieval and stop keywords used for irrelevant tweets filtering

Keywords	Stop Keywords
“suicide”, “suicidal”, “suic”, “self-harm”, “self-injury”, “self harm”, “self injury”, “hang myself”, “hung myself”, “kill myself”, “kills myself”, “killed myself”, “take my life”, “takes my life”, “want to die”, “wanted to die”, “wants to die”, “want death”, “wants death”, “wanted death”, “to be dead”	“bomb”, “suicide attack”, “suicide attacks”, “car attack”, “car attacks”, “suicide hotline”, “https://”, “http://”

### Tweets annotation

To identify tweets related to suicide and recognize the psychiatric stressors from tweets, two types of information were annotated. The first type of information is the label of the tweets. We annotated true suicide related tweets by choosing the label from *Positive*/*Negative*. *Positive* means the tweet is related to suicide or suicide ideation of the Twitter user (personal experience or feeling); tweets with the *Negative* label can be further categorized as 1) not related to suicide or suicide ideation, 2) the negation of suicide or suicide ideation (e.g. I don’t want suicide), 3) the discussion of suicide or suicide ideation of other people, 4) the news or reports, 5) other non-positive tweets. Sample tweets with positive and negative labels are illustrated in Table 2.

**Table 2:** Examples of tweets labeled with *Positive* or *Negative* in terms of relatedness to suicide

Positive	i don’t know why my dad always comments on how much i’m eating because it makes me want to die
	i’m tired of losing friends and people close to me cause of being suicidal
	i want to kill my self
	i’m in pain, wanna put ten shots in my brain i’ve been tripped by some things i can’t change suicidal
Negative	in the uk the biggest killer for men is suicide. Good job feminists ignoring their issues
	a dear friend of mine committed suicide with a shotgun two years ago
	i don’t say this lightly - hemingway’s life ended by suicide. His life was actually a loss
	these r not ur problems dear!! these r ur x bf’s commitng suicide

The second type of information is the mention of psychiatric stressors in tweets text. For the tweets annotated with label *Positive* (i.e., suicide-related), we further annotated the mentions of psychiatric stressors. Some examples of stressors are listed in Table 3.

**Table 3:** Examples of psychiatric stressors annotation from the suicide-related tweets. **Bold** refers to the annotated stressors

**job hunting** makes me want to commit suicide lmao	
honestly every time i think about me **getting pregnant** i wanna kill myself	
i just realized that i was completely **sexually assaulted** by some disgusting photographer and i want to fucking kill myself	
well i guess it’s too bad that i’m just **young adult without a degree** about to lose my **job** and probably planning my suicide afterwards	
**school** and **work** make me want to die everyday	

Here we divided this annotation process into two rounds: we first assigned *Positive/Negative* labels to 3263 tweets. We then trained a binary deep learning based classifier. We will introduce the classifier in the next section. We used this classifier to further select 3000 additional suicide related tweets with *Positive* labels that were relatively more precise than the original set. In the second round of annotation, we annotated the *Positive/Negative* labels as well as the mentions of psychiatric stressors on the new 3000 tweets. We leveraged the Clinical Language Annotation, Modeling, and Processing Toolkit (CLAMP) for the annotation process [24].

### CNN based binary classification to recognize suicide related tweets

**Fig. 2:**
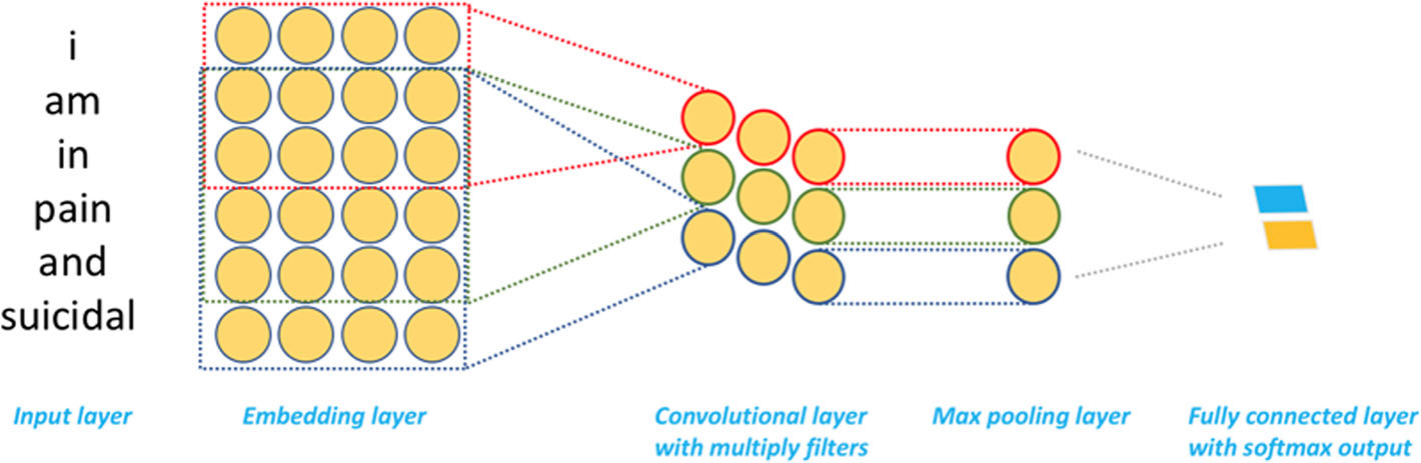
The architecture of CNN based binary classifier for suicide related labels prediction

As one deep learning based algorithm commonly used in various computer vision tasks [25], CNN also demonstrated excellent performance in many NLP tasks, including various text classification tasks [26–29]. We leveraged a classic CNN model for short text classification proposed by Kim et al. [26] to build the tweets binary classifier. We cleaned the tweets using the script from Stanford [30]. Then, we converted the tokens in each tweet to one-hot vectors and mapped the one-hot vectors to pre-trained GloVe Twitter embedding. The mapped embedding was used as the initial input feature to the CNN model. For the CNN model training, various filters were applied to generate the convolutional layers. We applied the max pooling strategy on the feature maps generated by different filters. We added dropout on the pooling layer to avoid overfitting. The pooling layer was connected to a fully connected layer with softmax output. The architecture of the CNN framework is shown in Fig. 2.

### RNN-based named entity recognition for stressors

**Fig. 3:**
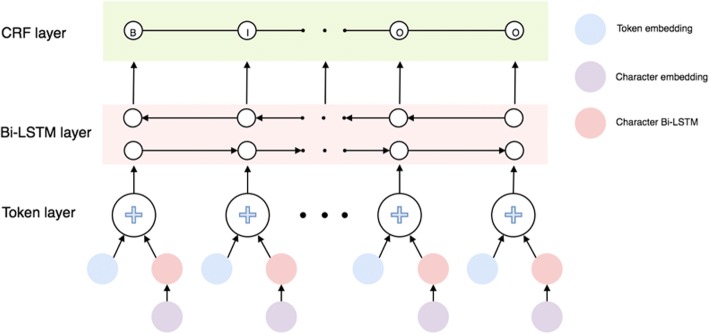
The architecture of the RNN framework for NER

We conceptualized the stressor recognition as a NER task and leveraged a state-of-art RNN framework proposed by Dernoncourt el al [31, 32] to extract the mentions of stressors. The architecture of the framework is shown in Fig. 3. The token embedding mapped each token in the tweets text to a token vector. The character embedding mapped the characters in each token to character vectors. The character Bi-LSTM took the character embedding at each time step as the input and outputs the summary of each character for each token. The token embedding was concatenated with the character LSTM output and then fed to the token Bi-LSTM layer together. On top of token Bi-LSTM, we applied a sequential CRF to jointly decode labels for the whole tweet.

### Transfer learning-based stressor recognition from tweets

Transfer learning has proven to be an effective technique to improve the performance on a target task with limited annotation data, by using some knowledge learned from a source task [33, 34]. Instead of training the model for a target task from a completely blank network, transfer learning can re-use all or some of the parameters trained from a source task. In our previous studies on extraction of stressors from clinical notes [8, 10], we created a dataset with stressors annotations from the psychiatric notes provided by the CEGS N-GRID 2016 challenge organizers [35]. The annotated dataset contained 946 sentences with stressors annotation. For the transfer learning based approach, clinical notes were used as the source domain to transfer stressor related knowledge to the target domain: Twitter.

### Experiment configuration

The core of the deep learning based framework in this study had two modules: a CNN based binary classifier to select suicide related tweets and a RNN based NER system to extract the mentions of stressors. The following experiments were performed for these two modules separately.

For the binary classifier, we trained the CNN model in two steps using two rounds of annotations, respectively. We first trained a CNN model on the first round of annotations with *Positive/Negative* labels. To improve the classifier performance on the unbalanced class distribution (623 *Positives*/3263 tweets), especially on the *Positive* class (suicide related), the training corpus was built with equal sample sizes of the *Positive* class and the *Negative* class (*Positive*: 498 tweets, *Negative*: 498 tweets); the evaluation corpus was built using the same distribution of origin tweets class (*Positive*: 125 tweets, *Negative*: 652 tweets). We further used the best classifier on the evaluation corpus to select another 3000 candidate suicide related tweets that potentially have a high proportion of *Positive* labels for the second round of annotation. The labeled tweets in the later 3000 candidate tweets were divided into training, validation and testing sets with a proportion of 7: 1: 2.

The CNN based classifier was trained and evaluated based on the three sets. We used the GloVe Twitter embedding to initialize the embedding layer of CNN and compare the performance using dimensions at 50, 100, and 200 respectively. In order to confirm the superiority of the CNN model, we also evaluated several traditional machine learning algorithms, including Extra Trees (ET), Random Forest (RF), Logistics Regression (LR) and Support Vector Machine (SVM) with Radial Basis Function (RBF) as the kernel, as well as a Bidirectional Long Short-Term Memory (Bi-LSTM) classifier. All the algorithms use GloVe Twitter embedding (dimension 50) as the input. The traditional classification algorithms take the average of the word vectors of the tweet text as the input feature. We evaluated the performance of the classifiers using standard metrics, including precision, recall and F-measure.

For the RNN based NER, we performed the following experiments:

1. To demonstrate the superiority of the RNN model, we used classic Conditional Random Fields (CRF) as the baseline model. We leveraged CLAMP for implementation [24]. Typical features for named entity recognition in CLAMP were employed including lexical features (e.g., bag-of-word, cases, prefix/suffix/stem patterns), syntactic features (e.g. Part of Speech tags), context features (e.g., n-grams), distributional representation of words (e.g., brown clustering, word embedding), and domain knowledge features (e.g., semantic types in UMLS), etc.
2. Performance comparison of different GloVe Twitter embedding dimensions at 50, 100, and 200 on stressor recognition tasks. Performance of different embedding dimensions were evaluated and reported using standard metrics, including precision, recall and F-measure, based on exact match (same entity boundary) and inexact match (overlap in entity boundary) respectively.
3. Performance comparison between using transfer learning strategy and training on Twitter data only for stressor recognition. For the transfer learning approach, clinical notes were used as the source domain to transfer stressor related knowledge to the target Twitter domain. The tweets with *Positive* label in the second round of annotation were divided into training, validation and testing set with a proportion of 6: 2: 2. Specifically, the following experiments were conducted:
  - Performance comparison using different sizes of annotated tweets for training in the transfer learning strategy and training on Twitter data only. We evaluated the performance of using different training data sizes from 5, 10, 20, 30, 40, 50 to 60% (all training data) respectively. The validation and testing datasets were kept with the same configuration consistently. We reported the standard metrics, including precision, recall and F-measure, based on exact match and inexact match respectively.
  - Performance comparison of transferring pre-trained parameters up to different layers of the RNN framework, from token embedding layer, character embedding layer, character LSTM layer, token LSTM layer, to fully connected layer to the final CRF layer.

## Results

### Annotation of suicide related tweets and psychiatric stressors

In the first round of annotation, we assigned the *Positive/Negative* labels to 3263 tweets. Among these tweets, only 623 tweets were annotated as *Positive* (suicide related). We trained the binary classifier (P: 0.66, R: 0.79, F: 0.72) on this annotated dataset and further selected 3000 tweets for the second round of annotation. Among these 3000 tweets, 1985 tweets were annotated as *Positive*, and 2162 stressor entitles were annotated in the *Positive* tweets.

### Suicide-related tweet classification

Table 4 lists the performance of suicide-related tweets classification, using the CNN based algorithm and GolVe Twitter embedding features of different dimensions. As shown, the *Positive* type had a high recall (0.90 as the optimal). It also achieved an overall F-measure sufficient (0.83 as the optimal) for practical applications.

**Table 4:** Experimental performance of suicide-related tweets classification, using the CNN based algorithm and word embedding features of different dimensions. **Bold** number denotes the largest number in that row

		D = 50	D = 100	D = 200
Precision	Positive	0.78	0.76	**0.79**
	Negative	0.69	**0.70**	0.65
Recall	Positive	0.88	**0.90**	0.84
	Negative	0.51	0.45	**0.56**
F-1 measure	Positive	**0.83**	0.82	0.81
	Negative	0.59	0.55	**0.60**

Table 5 lists the comparison of the CNN based algorithm with traditional machine learning algorithms as well as the Bi-LSTM model, using GloVe Twitter embedding (dimension 50) as the input. As shown, the CNN model led the performance in *Positive* type, *Negative* type and the overall accuracy. Bi-LSTM was second to the CNN model.

**Table 5:** Performance comparison of the CNN model with other algorithms. SVM: Support Vector Machine; ET: Extra Trees; RF: Random Forest; LR: Logistics Regression; Bi-LSTM: Bi-directional Long Short-Term Memory. **Bold** number denotes the largest number in that row

		CNN	SVM	ET	RF	LR	Bi-LSTM
Precision	Positive	**0.78**	0.7	0.69	0.69	0.7	0.73
	Negative	0.69	**0.72**	0.58	0.5	0.67	0.65
Recall	Positive	0.88	**0.96**	0.94	0.88	0.94	0.9
	Negative	**0.51**	0.21	0.17	0.24	0.23	0.37
F-1 measure	Positive	**0.83**	0.81	0.79	0.77	0.8	0.81
	Negative	**0.59**	0.33	0.27	0.33	0.34	0.47
Accuracy		**0.74**	0.703	0.689	0.665	0.697	0.72

### Psychiatric stressor extraction

#### Performance of RNN-based stressor recognition

In our pilot work, we evaluated the impact of different types of word embedding for stressors recognition. We first set embedding dimension at 100 and compared GloVe Twitter embedding, GloVe embedding and MIMIC embedding. GloVe Twitter embedding achieved best exact match and inexact match F-measure at 53.25 and 65.46% respectively. As a result, we chose GloVe Twitter embedding for further analysis. We further evaluated the impact of dimensions of the word embedding. The dimension at 100 achieves best exact match F-1 measure (53.25%), while the dimension at 50 achieves best inexact match F-1 measure (67.94%). Although the CRF baseline model led the precision of stressor recognition, however, as we can observe, the RNN based models greatly improved the recall, thus achieving better F-1 measure. The details of metric scores are in Table 6.

**Table 6:** Experimental performance metric of stressors recognition on different types of word embedding for both exact and inexact match. CRF: Conditional Random Fields. **Bold** number denotes the largest number in that column

	Precision		Recall		F-1 measure	
	exact	inexact	exact	inexact	exact	inexact
GloVe Twitter 50	0.4868	0.6843	0.4765	**0.6745**	0.4816	**0.6794**
GloVe Twitter 100	0.5822	0.7123	0.4906	0.6057	**0.5325**	0.6546
GloVe Twitter 200	0.5248	0.6808	**0.4977**	0.6484	0.5108	0.6642
CRF	**0.600**	**0.784**	0.398	0.572	0.478	0.661

#### Performance of transfer learning -based stressor recognition

Initialized with GloVe Twitter embedding with dimensions of 100, the best model trained on clinical notes achieved precision of 53.08%, recall of 47.51% and F-1 measure of 50.14% on the clinical notes validation set. We transferred parameters up to different layers from this model to initialize the RNN model training on Twitter dataset.

**Fig. 4:**
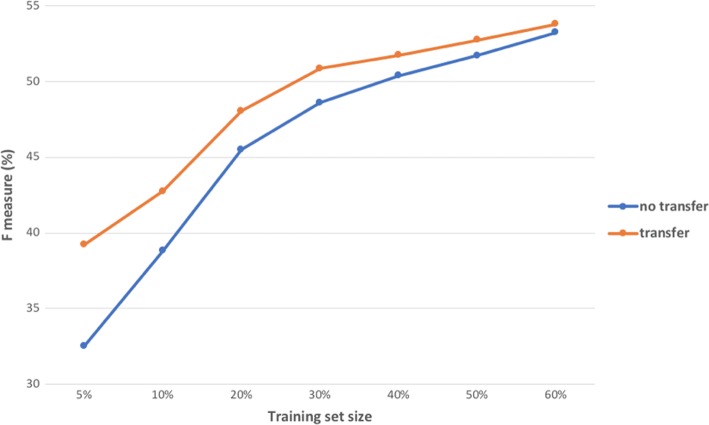
Impact of transfer learning on the size of training data measured on F-1 measure by exact match

We first investigated the impact of varying training set sizes of the Twitter dataset (see Fig. 4). For both learning strategies, F-measure improved as more training samples were used. Transfer learning improved the F-measure over training with Twitter data only, though the improvement diminished as more training samples were used. This phenomena is consistent with transfer learning on other NER tasks [34]. Compared to training with Twitter data only, transfer learning strategy can save on the number of annotations to achieve the same level of performance. For example, as shown in Fig. 4, transfer learning using 30% of the Twitter training data can achieved a higher F-measure than the baseline strategy where 40% of the training data was used.

**Fig. 5:**
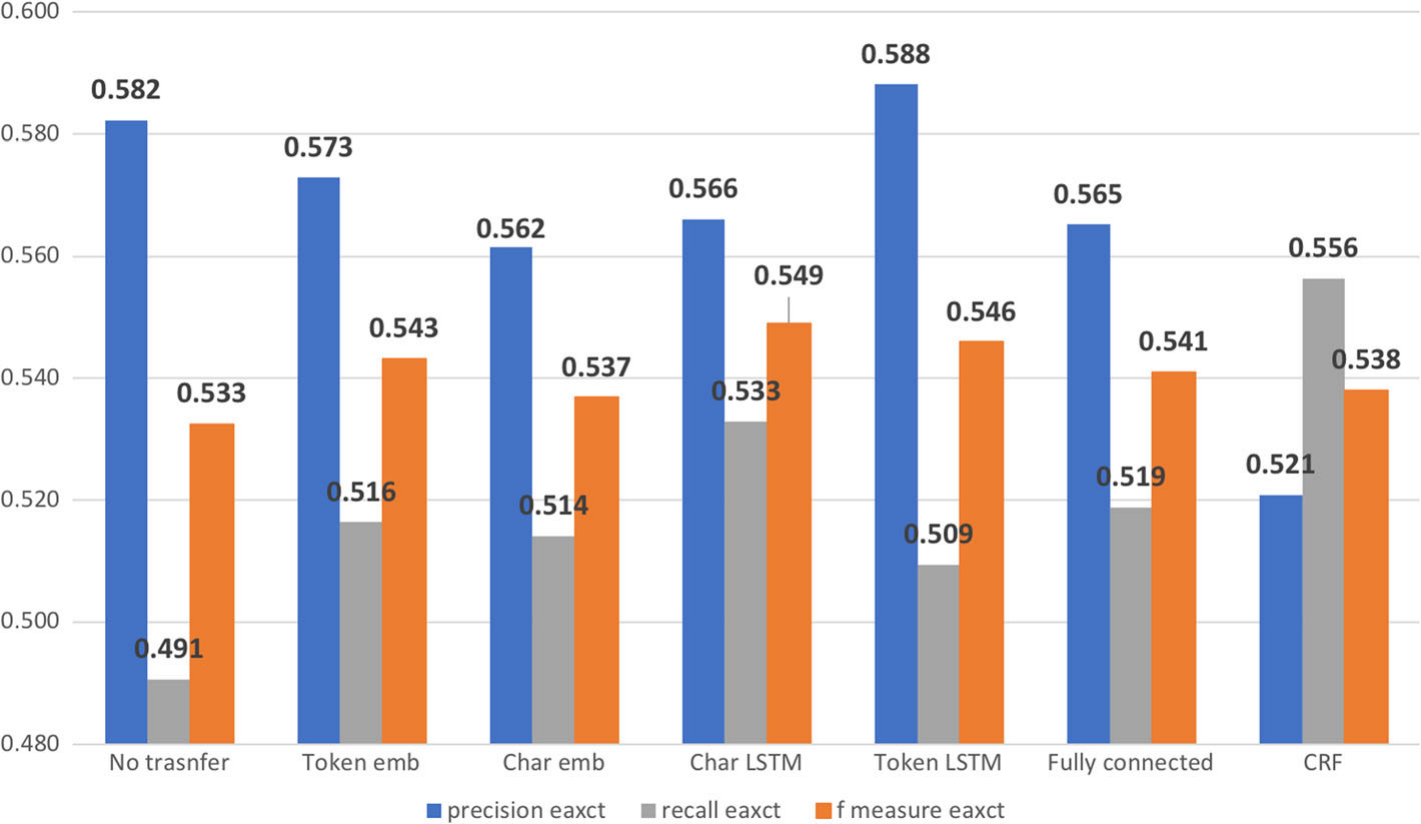
Impact of transferring the parameters up to each layer of the RNN model for stressors recognition measured by exact match

**Fig. 6:**
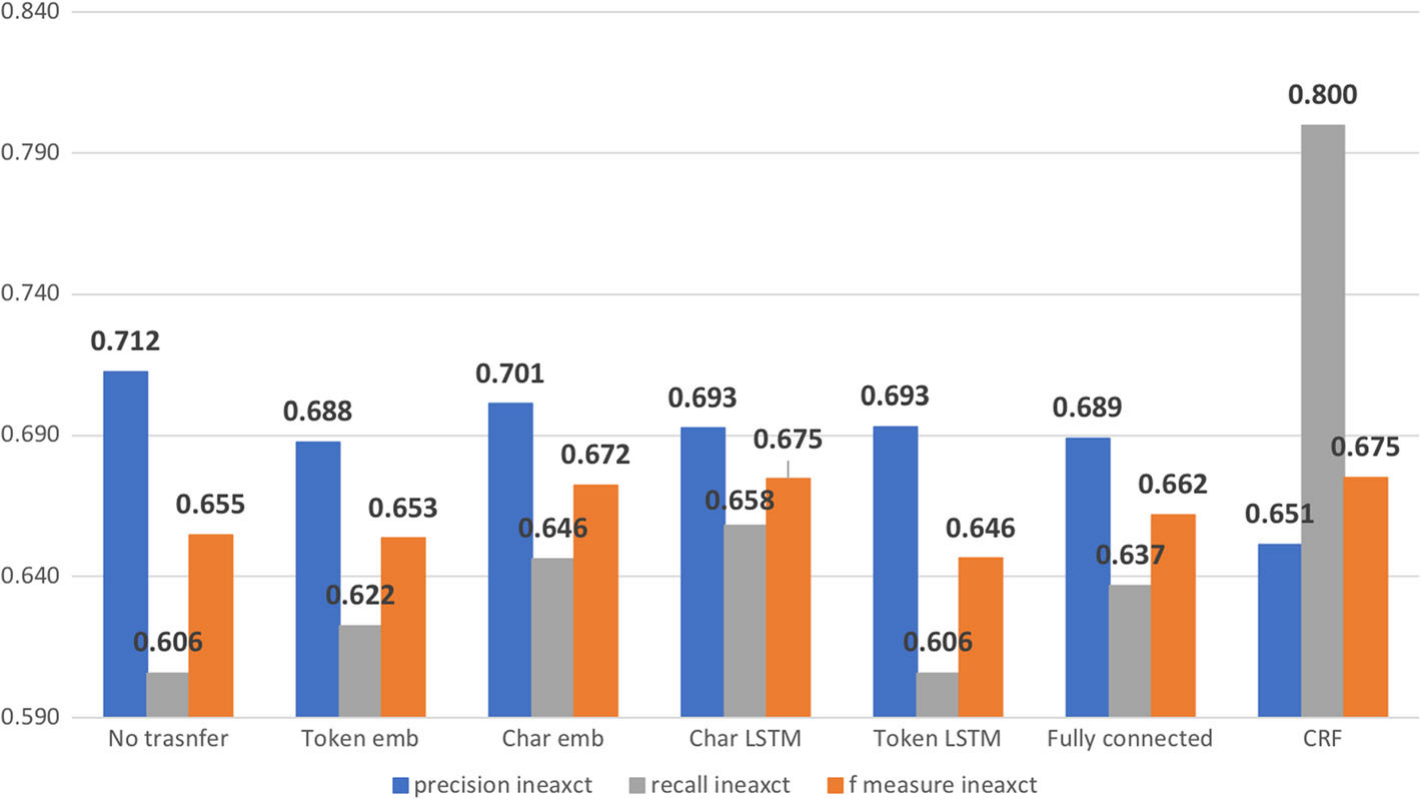
Impact of transferring the parameters up to each layer of the RNN model for stressors recognition measured by inexact match

**Fig. 7:**
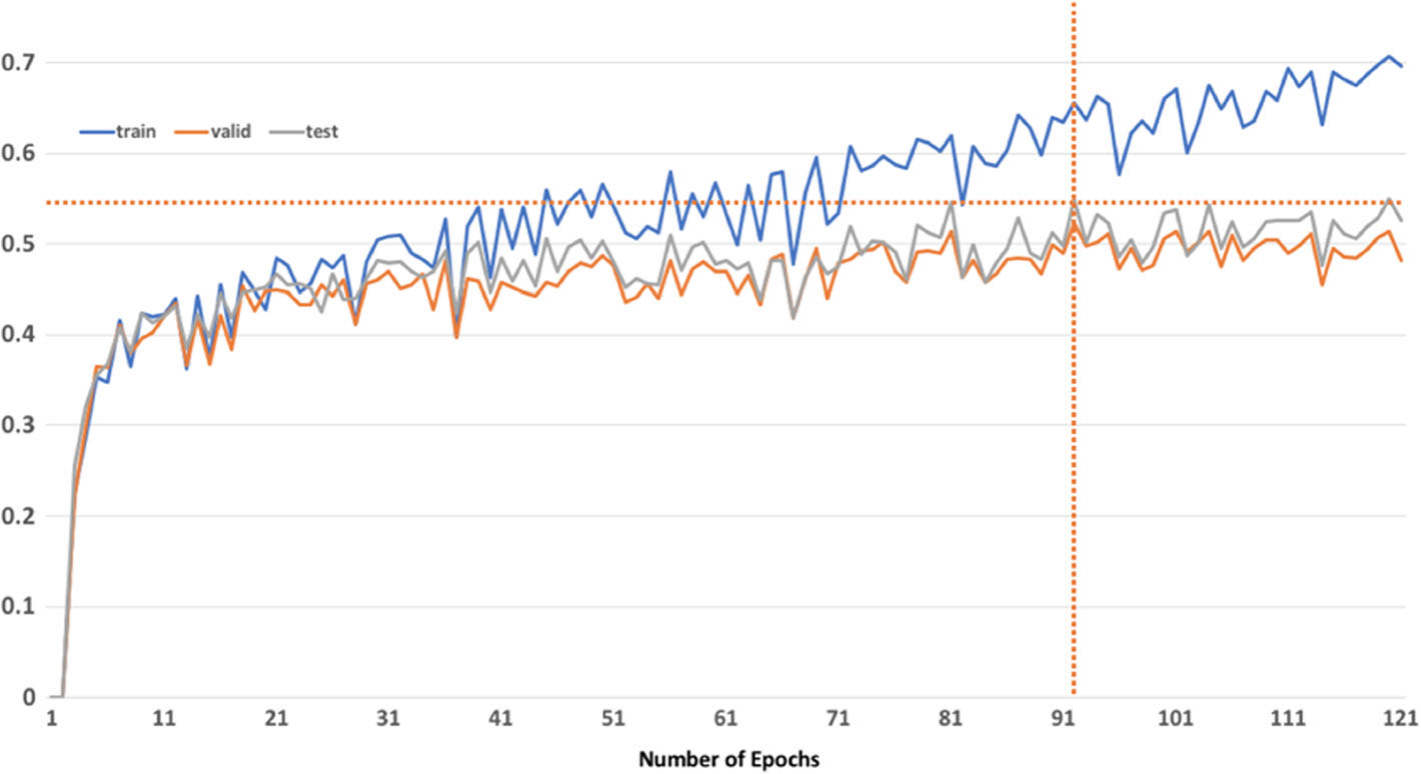
Exact match F-1 measure by each epoch from the model transferring up to character LSTM layer

Figures 5 and 6 show the impact of transferring the parameters up to each layer of the RNN model measured by exact match and inexact match respectively. For exact match, transferring the layers up to character LSTM achieved best F-measure at 54.9% (see Fig. 7), compared with 53.25% achieved by the model without transfer learning. Transferring all layers could also increase the F-measure, but not as much as transferring some lower layers only. As for inexact match, transferring the layers up to the character LSTM layer achieved a F-measure very close to transferring all layers (67.47 to 67.51%), out-performing by ~ 0.02 the model without transfer learning (F-measure, 65.46%).

## Discussion

In this paper, we proposed and evaluated a systematic pipeline to extract psychiatric stressors for suicide and suicidal ideations from Twitter. This pipeline had multiple steps: 1) Curation of a precise suicide related Twitter corpus by using keywords to collect and filter, and a deep learning based classifier to further select out suicide related tweets. This deep learning based classifier achieved a good F-measure at 83%, which is sufficient for practical application. 2) Leverage a state-of-art RNN based framework to extract stressors from suicide related tweets. This framework achieved the best F-measure by exact match at 53.25%. We also investigated the impact of transfer learning from clinical notes for the Twitter corpus. We found that transfer learning can achieve the same level of performance while reducing the annotation cost of tweets, in comparison with using only the Twitter data for training. For this RNN based framework, we found that transferring the parameters up to the character LSTM layer achieved the best F-measure by exact match (54.9%), 1.65% higher than the best model without transfer learning (F-measure: 53.25%).

To our best knowledge, this is the first effort to extract psychiatric stressors from Twitter data using deep learning based approaches. Compared with lexicon based text analysis, which often introduces much noise, this framework will facilitate more precise analysis. The deep learning based framework also saves great effort on feature engineering, compared to the conventional machine learning based approaches that require many hand-crafted features. A common limitation of machine learning based Twitter data analysis is the highly imbalanced dataset [28, 36]. We mitigated the impact of imbalanced distributions of classes and entities by building a precise suicide related Twitter corpus using multiple steps. Using state-of-art deep learning based approaches and the transfer learning strategy, our pipeline o achieved a reasonable performance on both suicide related tweets prediction as well as psychiatric stressors extraction.

Certain limitations remain for our study. The major limitation of this study is the lack of ground truth data for the exact mental health status of the Twitter users. The annotations were based on the contents of the tweets. As tweet contents may not always reflect the true mental health status, our dataset may include some false positive suicide related tweets. In addition, due to the limitation of data collection, the current analysis is only based on a single tweet. The lack of context of tweets may introduce inexact interpretations of the contents. There also still exists room for performance improvement on stressor recognition task. The most common type of prediction error in stressor recognition is the boundary issue. As illustrated in Table 7, causes of prediction errors also include (1) missing annotations in the gold standard, (2) the lack of negation detection and (3) mistakenly predicting high-frequent mentions of stressors within a wrong context.

**Table 7:** Common types of prediction errors. **Bold**: annotated entity; Underline: predicted entity

Boundary	• when the bottom of my **foot inches** i want death. • **allen high** **school** makes me want to kill myself for not allowing me to get out of a 2nd math class (that i don’t need) knowing i work • **hormonal** **birth control** made me suicidal and acne-ridden! there’s no winning against the beast within me
Annotation error: missing annotation	• harassment is not a thing you should have fun with. it almost killed me yesterday.
Negation	• im confident in my maths but **phys** makes me want to die i want
Entity in wrong context	• gonna kill myself after work today. Cant take the **bullying** no longer.
False negative	• when will my **best friend** stop telling me every little things she does with her boyfriend. i want death • i’d kill myself to make **everybody** pay

This study obtained a modest performance (exact match F-measure: 54.9%) on the stressors recognition task using deep learning based methods. In fact, Twitter NER tasks appear to be more challenging than NER tasks in other domains. For example, in the recent two Twitter NER challenges [37, 38], most of the state-of-art systems achieved F-1 measure between 40 to 60% on various entity recognition tasks, including *person*, *location,* etc. Considering stressors recognition is an even more complicated problem (i.e. various lengths of boundary, sparseness of expression patterns), we posit that our result is comparable to the state-of-art performances achieved so far.

As for the future work, we’re in the process of preparing a large-scale suicide related Twitter dataset. We will access the user level information by analyzing the historical tweets instead of the single tweet without any context. Ground truth data showing the mental health status of the users will be acquired by crowdsourcing or linkage to the users’ electrical health record (EHR) data. To improve the performance of the stressors recognition, we will further refine the quality of the gold standard annotation. Moreover, the deep learning based NER systems will be augmented with domain knowledge based and context based rules for further performance improvement.

## Conclusions

Few automated approaches have been proposed to extract psychiatric stressors from Twitter, mainly due to (a) the lack of annotated corpora that are time-consuming and costly to build, and (b) the inherent linguistic difficulties that stressors present beyond well-defined clinical concepts such as diseases. To our best knowledge, this is the first and comprehensive effort to extract psychiatric stressors from Twitter data using deep learning based approaches. We’ve build an annotated Twitter corpus on suicide related stressor recognition. We also performed extensive experiments to justify the use of the approaches presented. The comparison to traditional machine learning algorithms showed the superiority of deep learning based approaches. Our methods demonstrated good performance at identifying suicide-related tweets with a F-1 measure of 83%. In addition, stressor recognition obtained the best F-1 measure of 53.25% by exact match and 67.94% by inexact match. Moreover, transfer learning strategy was found to further improve the performance and show potential for saving annotation effort. The results indicate the potential to use deep learning based methods for automated stressor recognition in social media.

## Abbreviations

Bi-LSTM: Bidirectional long short-term memory
CLAMP: Clinical language annotation, modeling, and processing toolkit
CNN: Convolutional neural networks
CRF: Conditional random fields
ET: Extra trees
LR: Logistics regression
LSTM: Long short-term memory
NER: Named entity recognition
NLP: Natural language processing
RBF: Radial basis function
RF: Random forest
RNN: Recurrent neural networks
SVM: Support vector machines
